# Regional and cannabis-related differences in prefrontal multiscale entropy of resting-state EEG

**DOI:** 10.1016/j.cnp.2026.02.003

**Published:** 2026-02-19

**Authors:** William T. Creel, Colleen A. Brenner, Richard E Hartman

**Affiliations:** Department of Psychology, Loma Linda University, Loma Linda, CA 92354, USA

**Keywords:** Entropy, Electroencephalography, Prefrontal cortex, Cannabis, Neurophysiology

## Abstract

**Objective:**

Entropy analysis of electroencephalographic (EEG) data provides insight into the complexity of neural activity. This study primarily examined whether cannabis use frequency is associated with alterations in multiscale entropy (MSE) in the prefrontal cortex (PFC) and mapped baseline MSE differences across cortical regions.

**Methods:**

Resting-state EEG was collected from 57 adults: non-users (n = 18), low-frequency users (≤ 1x/week; n = 24), and frequent users (≥ 2x/week; n = 15). MSE values were binned into fine, medium, coarse, and very-coarse scale ranges. Linear mixed-effects models assessed group × scale bin interactions in the PFC and regional differences among lobes, irrespective of cannabis use.

**Results:**

Entropy increased with coarser scales in all groups, but the slope was significantly flatter in frequent users. From the medium bin onward, their PFC scale–entropy slope was ∼ 0.12 bits lower than in non-users, widening to ∼ 0.16 bits at very-coarse scales (FDR-corrected q < 0.012). Across all participants, the PFC exhibited lower MSE than parietal, occipital, and temporal lobes, with regional gaps expanding at coarser scales.

**Conclusions:**

The PFC exhibits intrinsically reduced signal complexity compared with other cortical regions, with further attenuation in frequent cannabis users.

**Significance:**

MSE captures regional cortical dynamics in resting EEG and detects cannabis-related reductions in prefrontal signal complexity at longer temporal scales.

## Highlights

• Frequent cannabis use is linked to reduced prefrontal complexity at coarse scales.
• Prefrontal cortex shows lower multiscale entropy than other regions in healthy adults.
• Multiscale entropy reveals scale-dependent cortical organization beyond power.

## 1 Introduction

A growing body of research has identified entropy analysis of electroencephalographic (EEG) data as a valuable indicator of brain function (Keshmiri, 2020, Chung et al., 2016, Garrett et al., 2013). Entropy, a concept that originated within thermodynamics, has been adopted to the study of biological systems. In its broadest form, entropy refers to the degree of disorder, randomness, or unpredictability in a system. Entropy has emerged as a powerful tool for quantifying the complexity of brain function and information processing with proven applications across multiple fields of neuroscientific research (Chung et al., 2016). Brain signal complexity, as quantified by entropy measures, varies systematically across development, aging, and neurological/psychiatric conditions, with both reduced and excessively high entropy associated with altered brain dynamics (Keshmiri, 2020, Shyu et al., 2015, Cacciotti et al., 2024). Recent work indicates that intermediate levels of brain entropy are associated with better cognitive performance and creativity, whereas deviations in either direction may reflect suboptimal dynamics (Xin et al., 2024, Shi et al., 2020).

Costa et al. (2002) introduced multiscale entropy (MSE) analysis, a method for quantifying the complexity of physiologic time series data across multiple temporal scales, to address limitations in traditional single-scale entropy algorithms. Whereas single-scale measures such as sample entropy or Lempel-Ziv complexity can yield apparently contradictory results in biological systems, MSE evaluates complexity separately at fine and coarse temporal scales, which are thought to reflect localized and more distributed information processing, respectively (Costa et al., 2005, Courtiol et al., 2016).

Although traditional spectral analysis provides robust frequency-domain insights into oscillatory brain activity, MSE complements this approach by quantifying the temporal complexity of EEG signals across multiple timescales, as evidenced in healthy populations and various neurological conditions (Cofré and Destexhe, 2025, Cacciotti et al., 2024, Angsuwatanakul et al., 2020). This complementarity justifies the inclusion of MSE alongside established methods, such as a Fast Fourier Transform (FFT), and may enhance clinical and research decision-making.

Previous EEG studies using single-scale Lempel-Ziv complexity have reported increased global signal complexity after acute THC administration in occasional users (Cortes-Briones et al., 2015) and in cannabis-dependent individuals (Laprevote et al., 2017). However, neither study screened for psychiatric comorbidity, which is a major confound given that 60–80% of individuals with frequent or dependent cannabis use meet criteria for at least one additional psychiatric disorder (Hasin et al., 2016, Volkow et al., 2014), many of which are themselves associated with altered EEG complexity (Shyu et al., 2015, Molina et al., 2020). The present study therefore uses MSE to examine regional complexity in frequent cannabis users while acknowledging psychiatric comorbidity as a potential confound and including schizotypal personality questionnaire (SPQ) scores to account for variance related to psychosis-spectrum traits.

Recent work also demonstrates that resting-state entropy varies systematically across cortical regions and networks (Nezafati et al., 2020, McDonough and Nashiro, 2014), with flexible coupling between frontoparietal and default-mode regions linked to adaptive function (Safron et al., 2022, Chen et al., 2022). Coarse-scale entropy in particular is thought to index longer-timescale integrative processes that are sensitive to neuromodulatory influences (Courtiol et al., 2016). Of particular interest is entropy in the prefrontal cortex (PFC), which is richly endowed with CB1 cannabinoid receptors (Fitzgerald et al., 2019).

The primary aims of the present study therefore were to determine whether frequent cannabis use is associated with altered prefrontal MSE, particularly at coarse temporal scales and to characterize baseline differences in MSE across cortical lobes in healthy adults. We hypothesized that frequent cannabis users would show reduced MSE at coarse temporal scales compared to non-users and low-frequency users. Additionally, we conducted exploratory analyses of prefrontal spectral power using FFT across canonical frequency bands to assess potential complementary alterations in oscillatory power profiles across groups and regions, as well as frontal-parietal multiscale cross-entropy (XMSE) to examine interregional complexity dynamics.

## 2 Methods

### 2.1 Data acquisition

Resting-state electroencephalogram (EEG) data was collected from 57 participants (60.7% female) aged 18–48 (mean age: 22.66 ± 7.75 years). The subjects in the dataset identified as Caucasian (54.1%), Asian (39.3%), Hispanic (3.3%), Black (1.6%), and Aboriginal (1.6%), and were further categorized based on their reported cannabis consumptions patterns: non-users (n = 18), low-frequency users (≤ 1x/week, n = 24), and frequent users (≥ 2x/week, n = 15). All participants were screened for a psychotic disorder, with no participants self-reporting a historical diagnosis. However, the dataset did not include structured psychiatric assessments, and no standardized evaluations of mood, anxiety, or neurodevelopmental disorders were available. To partially account for symptom-related variance, all participants completed the SPQ, which captures psychosis-spectrum traits but does not substitute for a full psychiatric characterization. Accordingly, residual confounding related to psychiatric comorbidity cannot be excluded.

All procedures performed in this study involving human participants were in accordance with the ethical standards of the institutional research committee and with the 1964 Helsinki declaration and its later amendments. Ethical approval for the original data collection was obtained from the Institutional Review Board (IRB) at the University of British Columbia. Written informed consent was obtained from all participants. The archived EEG data used in this analysis were de-identified and all procedures conformed to the guidelines and regulations set forth by the approving committee.

Participants sat still in a dimly lit room for the duration of a single 3-minute, eyes-closed recording. This recording length has demonstrated high test–retest reliability (Popov et al., 2023, Duan et al., 2021). EEG data were recorded during standard daytime hours between 09:00 and 17:00 using 32 Ag/AgCL electrodes with a nose reference (Falk-Minow Services, Munich, Germany) and Neuroscan SYNAMPS recording system (Neuroscan, Inc., El Paso, TX, USA). Electrodes were placed in accordance with the international 10–20 system and obtained at a sampling frequency of 1000 Hz.

### 2.2 Preprocessing methods

EEG recordings were preprocessed using MNE, an open-source Python library designed for processing EEG data (MNE, v1.3.1; Gramfort et al., 2014). Recordings were band-pass filtered between 0.5 and 60 Hz to avoid contamination. The filtered time series was otherwise left largely unperturbed per recent preprocessing recommendations that suggest maintaining the integrity of the EEG data, especially in resting-state conditions (Delorme, 2023).

### 2.3 Multiscale entropy analysis

EntropyHub, an open-source toolkit for entropic time series analysis (Flood & Grimm, 2021), was used to calculate MSE in the EEG time series. To compute MSE, the time series was coarse-grained by averaging consecutive data points, such that the number of total data points is reduced by N/τ, where N is the number of data points and τ is the scale factor. Through this coarse-graining procedure, the new dataset can be represented by equation (1),

(1) $$y_{j}^{\left(\tau \right)}=\frac{1}{\tau }\sum_{i}\;=j\tau -\tau +1^{j\tau }x_{i},1\leq j\leq \frac{N}{\tau },$$

where τ is the scale factor and x_i_ is the original data point.

SampEn was then calculated across each consecutively coarse-grained time series to derive entropy at multiple scales. SampEn is determined according to equation (2),

(2) $$SampEn\left(m,r,N\right)=-\log\left(\frac{\sum A_{i}}{\sum B_{i}}\right)=-log\frac{A}{B},$$

where *m* is the embedding dimension and *r* is the tolerance threshold for acceptable matches (Angsuwatanakul et al., 2020).

Establishing uniform parameter values across studies ensures reliability of findings and their interpretations. Past studies have demonstrated that acceptable parameter values for SampEn include *m* = 2 and 0.15 ≤ *r* ≤ 0.20 (Costa et al., 2005, Castiglia et al., 2023). In the present study, the SampEn parameter values were set by default at *m =* 2 and *r* = 0.20 by EntropyHub (Flood and Grimm, 2021).

Time series were trimmed to 30 s to maintain consistency across analyses, and MSE was computed across 20 temporal scales. Richman and Moorman (2000) recommend that to achieve an accurate SampEn analysis, the minimum size for N must be at least 10*^m^-*20*^m^*. This is important when considering that N decreases with each subsequent scale increase. The EEG data in the present study was acquired at a sampling frequency of 1000 Hz, containing 30,000 data points across a 30 s epoch. Even at the 20th scale, the coarse-grained time series contains ample data points for an accurate SampEn analysis. All entropy values are expressed in bits.

### 2.4 Statistical analysis

All analyses were performed in Python 3.11 using pandas 2.2, NumPy 1.26, and the statsmodels 0.15 MixedLM routine for linear mixed-effects estimation. Visual checks of model assumptions and FDR-adjusted p-values were generated with the same toolchain. To ensure adequate statistical power for our primary linear mixed-effects model (PFC model), we conducted a Monte Carlo power simulation tailored to this design. The simulation was specified to detect a small group × scale bin interaction effect (β = 0.15), assuming α = 0.05 and an intraclass correlation of 0.50 for the 20 repeated measures within subjects. Based on 1000 simulated datasets, a total sample of N = 45 yielded 83.1% power, exceeding the conventional 80% threshold. Our achieved sample of N = 57 is therefore considered robust for detecting the hypothesized effects.

Electrodes were averaged into four lobes: frontal (Fp2, Fp1), parietal (Pz, P3, P4, P7, P8), occipital (Oz, O1, O2) and temporal (T7, T8). Multiscale entropy (MSE) values for scales 1–20 were binned into fine (1–5), medium (6–10), coarse (11–15) and very-coarse (16–20) ranges. Three complementary analytical approaches were employed to assess the signal properties: MSE was used to quantify intra-regional temporal complexity, FFT was used to quantify spectral power, and XMSE was employed to explore inter-regional complexity dynamics. All three methods utilized a Linear Mixed-Effects (LME) structure to test for interactions and group differences across their respective dimensions, with random intercepts for subjects:

1. PFC entropy analysis: To test our primary hypothesis while accounting for individual differences in traits typically associated with psychiatric conditions, we included the total SPQ score (SPQTotal) as a covariate. To test whether timescale-specific entropy effects were attributable to cannabis use frequency beyond the influence of these traits, and to control for age of first use (AgeFirstUse), a two-model procedure was applied to the frontal data.
  a. Model A (All Subjects, N = 57):

entropy ∼ group × scale_bin + SPQTotal × scale_bin + (1|subject)

b. Model B (Users Only, N = 39):

entropy ∼ group × scale_bin + AgeFirstUse + SPQTotal × scale_bin + (1|subject).

2. PFC spectral analysis: Explored differences between groups across the four canonical frequency bands (delta, theta, alpha, beta) using an identical structure to the entropy model.
  a. Model A (All Subjects, N = 57): 

LogPower ∼ Group × Band + SPQTotal × Band+ (1|subject)

b. Model B (Users Only, N = 39):

LogPower ∼ Group × Band + AgeFirstUse + SPQTotal × Band + (1|subject).

3. Regional entropy model: Assessed regional complexity differences, collapsing across all groups.

entropy ∼ lobe × scale_bin + (1|subject)

4. Regional spectral model: Assessed regional power differences, collapsing across all groups.

LogPower ∼ Lobe × Band + (1|subject)

5. Fronto-Parietal multiscale Cross-Entropy Model: Explored inter-regional complexity dynamics, while including SPQTotal only as a main-effect covariate.

XMSE ∼ Group × scale_bin + SPQTotal + (1|subject)

Fixed-effect significance for all models was assessed by Wald z tests. False discovery rate (FDR) correction using the Benjamini–Hochberg procedure was applied to post-hoc contrasts (e.g., band-wise or scale-bin comparisons). Omnibus fixed effects and planned covariates were evaluated using uncorrected Wald tests. Visual inspection of residual and random-effect diagnostics revealed no substantial deviations from model assumptions.

## 3 Results

### 3.1 MSE in cannabis users

**Fig. 1:**
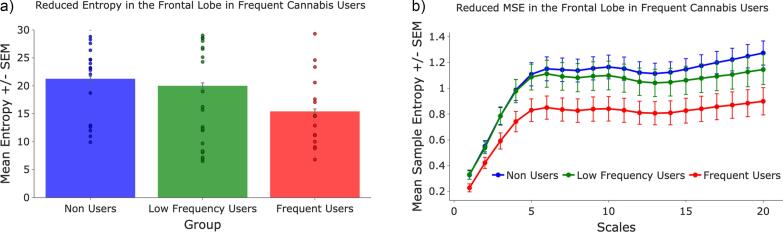
**A)** Mean PFC entropy collapsed across all 20 scales for each cannabis-use group. Individual participant values are over-plotted. Non-users show the highest overall entropy, frequent users the lowest, and low-frequency users occupy an intermediate, but statistically non-distinct relative to frequent users. **B)** MSE across groups in the frontal cortex. The steep rise from fine to medium scales is common to all groups; the non-user curve continues to climb, whereas the frequent-user curve plateaus, producing the group × scale interaction.

In our primary analysis (Model 1A), PFC entropy rose sharply from the fine to the medium bin (β ≈ +0.40), levelled off across the coarse bin (β ≈ +0.38), and exhibited a robust additional increase at the very-coarse bin (β ≈ +0.48; all q < 1 × 10^9^). Fine scale values did not differ among non-users, low-frequency users, and frequent users (all q > 0.29). However, a significant group × scale bin interaction emerged at the very-coarse bin, indicating that the shape of the entropy curve differed by group. Relative to non-users, frequent users showed a flatter PFC curve (Fig. 1B). This attenuation of the entropy increase was most robust at the very-coarse bin (β ≈ -0.16, q = 0.012) and present as a trend at the medium (β ≈ -0.12, q = 0.062) and coarse (β ≈ -0.13, q = 0.062) bins after FDR corrections. These findings were robust when controlling for individual differences; the group × scale bin interaction remained significant even after accounting for timescale-specific effects of schizotypy traits (SPQTotal), which was not itself a significant predictor of the MSE curve shape (SPQTotal × scale_bin, all q > 0.985). Low-frequency users did not differ significantly from non-users at the medium and coarse timescales (all q ≥ 0.30), although a trend toward reduced entropy was observed at very-coarse scales (β = –0.11, q = 0.062). A follow-up analysis limited to the cannabis user groups (Model 1B) found that age of first use was also not a significant predictor of MSE (p = 0.336).

### 3.2 FFT analysis in cannabis users

In the spectral analysis (Model 2A), PFC spectral power showed a robust decrease from the Delta band (β = +1.55 relative to Alpha) to the Beta band (β = -1.00 relative to Alpha), consistent with a strong overall effect of frequency band (p < 0.001). The overall Group main effect did not reach conventional significance (p = 0.051), indicating that groups did not differ in average spectral power when collapsing across all frequency bands.

**Fig. 2:**
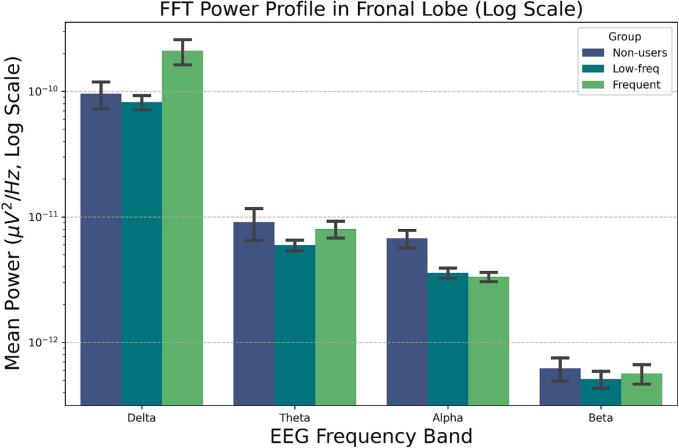
Mean spectral power across four bands, shown on a logarithmic scale. The LME revealed a significant Group × Band interaction, indicating a flatter spectral profile in non-users relative to frequent users (Delta power β = -0.431). However, follow-up contrasts failed to localize any specific band difference after FDR correction (all q > 0.400). Bars show the mean (± SE).

However, there was a significant Group × Band interaction (p = 0.034), indicating that the spectral shape differed by group. Relative to Frequent users, Non-users showed a flatter spectral profile, driven by reduced power in the Delta band (β = -0.431) and reduced power in the Beta band (β = -0.241), when both were compared against the Alpha reference band (Fig. 2).

These findings were robust when controlling for schizotypy traits; the Group × Band interaction remained significant, and the SPQTotal covariate was not itself a significant predictor of the spectral power curve shape (p = 0.966).

Follow-up LME contrasts were performed to localize the Group × Band interaction. After correcting for multiple comparisons, no group comparison in any frequency band remained statistically significant (all q > 0.400).

A follow-up analysis limited to the cannabis user groups (Model 2B) found that age of first use was also not a significant predictor of spectral power (all p > 0.115).

### 3.3 MSE across cortical lobes

**Fig. 3:**
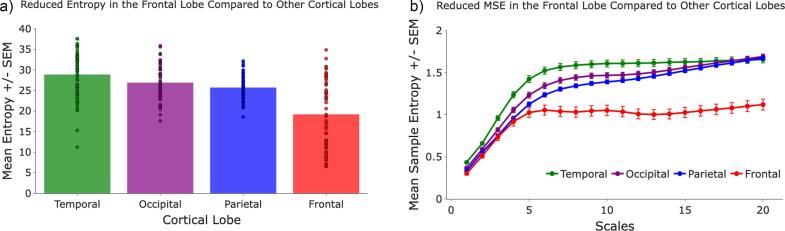
**A)** Collapsing across all 20 scales, the frontal cortex displayed the lowest mean entropy, with parietal, occipital, and temporal cortices progressively higher. **B)** The mixed-effects lobe × scale interaction revealed that frontal entropy plateaued by the medium range (scales 6–10)**,** whereas parietal, occipital, and temporal entropy continued to rise. By the very-coarse scales (16–20) those three posterior/temporal curves clustered together.

Across all participants, MSE rose monotonically from fine (scales 1–5) to very-coarse (scales 16–20) bins in every lobe (scale_bin main effect: β_medium = +0.35 bits, β_coarse = +0.32, β_very-coarse = +0.38; all q < 0.001; Fig. 3A). A strong lobe main effect showed that the frontal cortex carried the lowest entropy at the finest scales, with parietal (+0.05 bits), occipital (+0.12 bits) and temporal (+0.25 bits) regions progressively higher; all pairwise lobe contrasts remained significant after FDR correction (parietal q = 0.038; occipital and temporal q < 0.001). Critically, a significant lobe × scale bin interaction (p < 0.001) indicated that these regional gaps widened with scale: by the very-coarse bin, temporal entropy exceeded frontal entropy by ≈ 0.50 bits, while occipital and parietal lobes were approximately 0.44–0.49 bits higher (Fig. 3B). In contrast, frontal entropy plateaued early and remained the lowest across the entire scale range.

### 3.4 FFT across cortical lobes

**Fig. 4:**
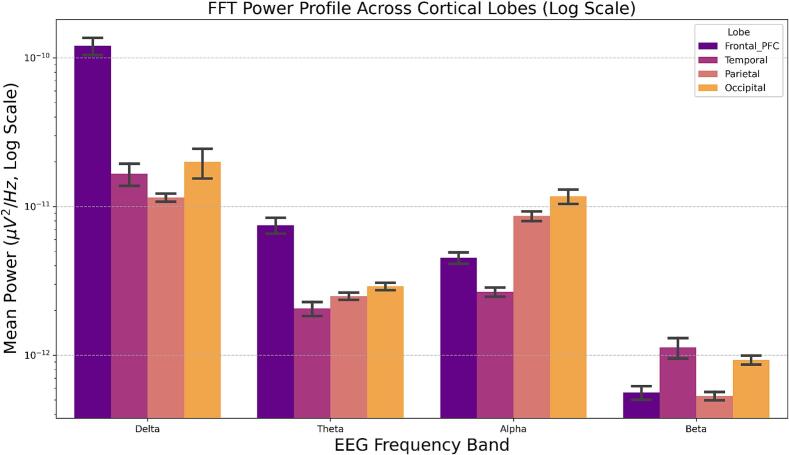
Spectral power by frequency band and cortical lobe. Across all participants, power differed by band and lobe (p < 0.001), following a canonical 1/f profile (delta highest, beta lowest). Regional differences were frequency-dependent, with higher power in occipital and parietal regions and lower power in temporal cortex.

Across all participants, spectral power differed significantly by frequency band and cortical region (main effects of Band and Lobe; both *p* < 0.001; Fig. 4). Collapsing across lobes, delta power was highest overall (β = +1.31), followed by theta (β = +0.19), while beta power was markedly reduced (β = -1.11, all *p* < 0.001), consistent with the canonical 1/*f* spectral profile. A significant lobe main effect indicated robust regional differences in spectral magnitude. Relative to frontal cortex, occipital (β = +0.33) and parietal (β = +0.18) regions showed higher power, whereas temporal cortex exhibited lower overall power (β = -0.25, all *p* < 0.001). Critically, multiple significant lobe × band interaction terms survived FDR correction (all q < 0.05 except occipital–beta), indicating that regional differences in spectral power were frequency-dependent and reflected distinct band-specific spectral profiles across cortical regions.

### 3.5 Frontal-parietal multiscale cross-entropy

**Fig. 5:**
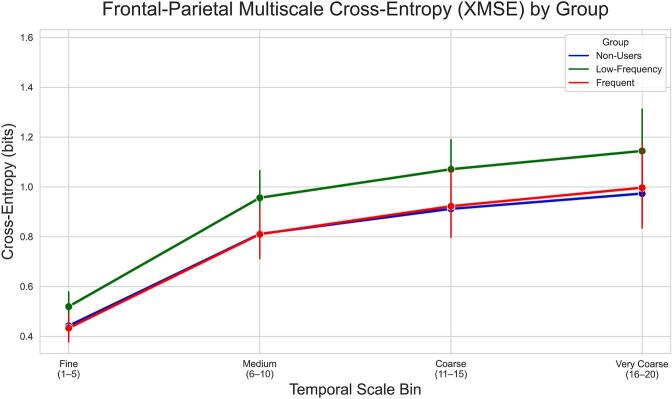
Exploratory frontal-parietal XMSE by group. XMSE increased across coarser scales (scale_bin main effect; Fine/Medium < Coarse, p < 0.05; Very Coarse trend, p = 0.131). Trend Group main effect: Low-Frequency > Frequent (β = 0.164, p = 0.078); Non-Users (p = 0.981 vs. Frequent). No Group × scale bin interaction (p > 0.32). Error bars: 95% CI.

Frontal-parietal XMSE increased across bins (main effect of scale_bin: Fine and Medium significantly lower than Coarse reference, p < 0.05; VeryCoarse trend p = 0.131). A marginal main effect of Group indicated a trend toward higher overall XMSE in Low-Frequency users relative to Frequent users (β = 0.164, p = 0.078; Fig. 5), with Non-Users not significantly different from Frequent users (p = 0.981). The Group × scale bin interaction was non-significant (all p > 0.32), suggesting group differences were consistent across scales. SPQTotal was not a significant predictor (p = 0.529).

## 4 Discussion

This study is the first to consider the impact of cannabis consumption on MSE levels within the brain, extending prior work investigating cannabis use on brain complexity using single-scale measures (Laprevote et al., 2017, Cortes-Briones et al., 2015). We observed a robust reduction in MSE within the PFC, independent of cannabis-use, relative to more posterior regions. Similar anterior-to-posterior gradients have been reported, but almost exclusively in pediatric or clinical cohorts (Angulo-Ruiz et al., 2023, Pelc et al., 2022). Our findings extend this pattern to a healthy adult sample, suggesting that lower PFC complexity may be a normative feature rather than one limited to developmental or pathological contexts. The MSE curve profiles we observed reveal a unique pattern that suggests the PFC exhibits significantly lower complexity at coarser temporal scales compared to other cortical regions. This lower complexity in the PFC may reflect region-specific specialization in resting-state signal organization, with more constrained dynamics at longer temporal scales potentially supporting stable integrative control processes (Carhart-Harris, 2018). Alternatively, lower complexity in the PFC may also be explained by developmental factors specific to this region of the brain. The PFC is well-known to be one of the last regions to fully mature (Kolk & Rakic, 2022). Accordingly, the PFC’s delayed maturation may partly account for its lower neural complexity relative to earlier-maturing cortical regions.

Notably, the further reduction in MSE levels in the PFC at coarse scales observed in frequent cannabis users, compared to non-users, supports our hypothesis and indicates that frequent cannabis use is associated with more constrained and less complex neural dynamics at temporal scales reflecting distributed information processing (Courtiol et al., 2016). The 0.16 bit reduction in very coarse-scale PFC entropy observed in frequent cannabis users suggests an attenuation of the normal complexity-scaling relationship. Mechanistically, this cannabis-related flattening at longer temporal scales may reflect reduced long-timescale integrative dynamics in prefrontal control systems, which could influence typical network interactions between frontal and posterior regions (Chen et al., 2022).

Our findings diverge from those of Cortes-Briones et al. (2015) and Laprevote et al. (2017), who reported increased single-scale complexity with acute THC and in cannabis dependence, respectively. These discrepancies are likely multifactorial including differences in population (chronic heavy users with probable higher comorbidity burden vs. screened healthy users), entropy metric (multiscale vs. single-scale), and spatial averaging all likely contribute.

In addition to our MSE analyses, exploratory spectral analyses were conducted to assess whether alterations in oscillatory power accompanied the observed entropy effects. No significant main effect of group was observed when collapsing across bands, indicating that cannabis use frequency was not associated with global differences in prefrontal oscillatory power. A significant Group × Band interaction indicated subtle differences in spectral profile shape, with non-users exhibiting a flatter spectrum characterized by relatively reduced delta and beta power compared to frequent users. However, follow-up band-specific contrasts did not survive correction for multiple comparisons (all q > 0.400), suggesting that these effects were modest and not localized to specific frequency bands. These findings indicate that traditional spectral analyses were relatively insensitive to group differences, whereas MSE revealed scale-dependent alterations in the temporal organization of PFC activity that differentiated frequent cannabis users from non-users, highlighting the complementary value of entropy measures. Future work should evaluate whether a combined spectral-entropy index provides incremental utility beyond either metric alone.

Regional FFT analyses revealed robust main effects of cortical region and frequency band, with higher overall power in occipital and parietal regions relative to frontal and temporal cortex, and significant lobe × band interactions indicating frequency-dependent regional spectral profiles. When interpreted alongside regional entropy analyses, these findings suggest that entropy provides complementary information to spectral power by capturing aspects of regional signal organization not fully reflected in oscillatory magnitude. Notably, temporal cortex exhibited the highest entropy despite the lowest overall power, underscoring the partial dissociation between power-based and complexity-based metrics.

Exploratory fronto-parietal XMSE analyses revealed the expected increase in inter-regional complexity across coarser temporal scales but did not yield robust cannabis-related effects. XMSE provides a non-directional measure of cross-regional signal irregularity and should be interpreted as a coarse index of fronto-parietal network coordination, reflecting shared versus differentiated temporal structure between frontal and posterior regions, rather than directed information flow. No significant Group × Scale interactions were observed, indicating that fronto-parietal XMSE was not significantly associated with cannabis use frequency. While beyond the scope of the present exploratory analysis, future studies may benefit from implementing network entropy flow analyses to better characterize inter-regional neural dynamics.

Several limitations must be acknowledged. Although we controlled for age of first cannabis use, schizotypal traits using the SPQ, and screened individuals for known psychotic disorders, the sample was modest in size and composed largely of healthy students, which limits generalizability. SPQ scores do not substitute for a full psychiatric evaluation and cannot account for comorbid conditions such as anxiety, depression, ADHD, or other psychiatric disorders, all of which are known to influence EEG signal complexity. Given that 60–80% of frequent cannabis users meet criteria for at least one additional psychiatric condition (Volkow et al., 2014; Hasin et al., 2016), residual confounding related to unmeasured comorbidity cannot be excluded. Additionally, the researchers did not collect neuropsychological or neurobehavioral data, preventing any direct linkage between the observed entropy reductions and cognitive performance or real-world functioning. The researchers also did not record the recency of last cannabis use or sleep quality the night before the recording. Although the study focused on habitual, frequent cannabis exposure rather than acute effects, the absence of these variables limits our ability to determine whether the observed entropy differences reflect stable neurophysiological adaptations associated with frequent use, transient effects of recent exposure, or residual influences related to sleep-dependent changes in cortical dynamics. Finally, group assignment relied on self-report, which is subject to bias. That said, the clear categorical separation between frequent users (≥2×/week) and complete non-users supports confident interpretation of the observed group difference in prefrontal MSE.

The present study focused on cannabis use frequency, but less is known about dose–response relationships on brain entropy. Future research should investigate the effects of low and high-dose cannabis consumption on entropy levels. Additionally, future studies should incorporate more robust psychiatric comorbidity measures and neuropsychological data to disentangle the specific effects of cannabis on neurophysiology and to determine whether entropy aberrations reflect differences in cognitive function.

## 5 Conclusions

In conclusion, this study contributes to an evolving field of research focused on entropy in EEG as a valuable indicator of brain function. The novel application of MSE analysis revealed intrinsically lower PFC complexity in healthy adults and an additional flattening of the prefrontal MSE curve in frequent cannabis users, most pronounced at coarse temporal scales. Notably, MSE was more sensitive than traditional spectral power analyses in differentiating frequent cannabis users from non-users. These findings underscore the complementary value of entropy-based measures relative to frequency-domain power metrics by capturing scale-dependent aspects of neural signal organization that are not fully reflected in oscillatory magnitude alone. Together, these results not only deepen our understanding of regional brain signal dynamics and the underlying neurophysiological effects of frequent cannabis consumption, but also highlight the practical utility of open-source MSE tools in EEG for furthering our understanding of brain function.

## 6 Disclaimer/Publisher’s Note

The statements, opinions and data contained in all publications are solely those of the individual author(s) and contributor(s) and not of MDPI and/or the editor(s). MDPI and/or the editor(s) disclaim responsibility for any injury to people or property resulting from any ideas, methods, instructions or products referred to in the content.

## Data availability

The datasets generated and/or analyzed during the current study are available in the GitHub repository, https://github.com/Tcgoalie29/MSE_Cannabis_Data/tree/main.

## Funding

This research did not receive any specific grant from funding agencies in the public, commercial, or not-for-profit sectors.

## Declaration of Competing Interest

The authors declare that they have no known competing financial interests or personal relationships that could have appeared to influence the work reported in this paper.
